# The reinvention of potato

**DOI:** 10.1038/s41422-021-00542-5

**Published:** 2021-08-23

**Authors:** Martin Mascher, Murukarthick Jayakodi, Nils Stein

**Affiliations:** 1 Leibniz Institute of Plant Genetics and Crop Plant Research (IPK) Gatersleben, Seeland, Germany; 2 German Centre for Integrative Biodiversity Research (iDiv) Halle-Jena-Leipzig, Leipzig, Germany; 3 Center for Integrated Breeding Research (CiBreed), Georg-August-University Göttingen, Göttingen, Germany

**Keywords:** Plant molecular biology, Genomic analysis

**True-seed potato has been the holy grail of potato breeding for decades; genetic improvement, notably resistance breeding, has lagged behind in this vegetatively propagated, polyploid staple crop. A recent study by Zhang et al. in** ***Cell*** **shows how genomics can greatly expedite the transition from tuber to seed crop and boost yields by growing F1 hybrids of custom-made inbred lines developed from plant genetic resources.**

“Mom” and “Dad” are the first words a child learns, and it is understood that mother and father are separate entities. Most plant species reproduce sexually like us, but many of them can self-fertilize so that their offspring have only one parent. Repeated self-fertilization leads to inbreeding, i.e, the fixation of all alleles in homozygous state. Inbreeding has been favored by early farmers, presumably because it helped fix and maintain recessive domestication traits, most of which, such as loss of seed or fruit dehiscence and adaptation to new photoperiod regimes, involved novel, recessive mutations.^1^

**Fig. 1:**
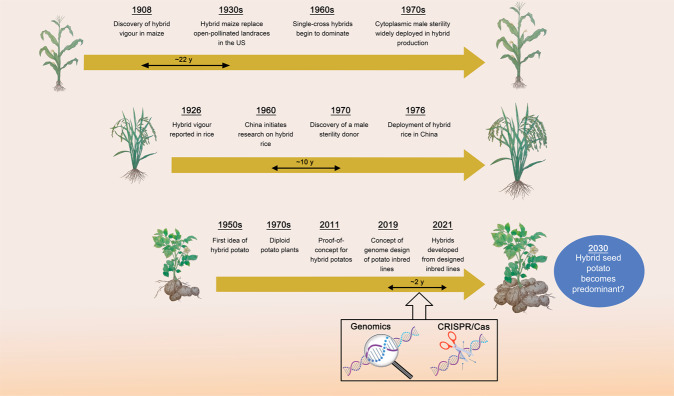
Time lines of the development of hybrid breeding in maize, rice and potato. It can take scientists decades to set up all the prerequisites for hybrid breeding and to translate them into applied breeding. After the discovery of diploid potatoes in the 1970s, progress towards hybrid potato has been stalled for decades. With the help of genomics, Sanwen Huang and co-workers were able to develop plant genetic resources into heterotic inbred lines within two years. The examples of rice and maize show that once all the tools are in place, hybrids take over rapidly. We wonder whether we will eat French fries from hybrid potato by 2030.

Many of the world’s leading crops are inbreeders, such as rice, wheat, and soybean. However, geneticists have known for more than a century that the offspring of two inbred lines outperforms its parents, often to quite a spectacular degree. The reasons for this *hybrid vigor* (also known as heterosis) have remained elusive, but may include the complementation of recessive deleterious alleles and the additive action of dominant beneficial alleles.^2^ Plant breeders have combined the best of both worlds: rapid and durable fixation of alleles by inbreeding and heterosis by judicious selection of heterotic groups of inbred lines to pair in crosses (Fig. 1). The six-fold yield increases in maize in the 20th century are in large part due to the development of hybrid breeding to supersede open-pollinated landraces.^3^ The development of hybrid rice by the late Longping Yuan made him a national hero in China and helped safe-guard global food security.^4^

It is obvious then to ask: can we turn all crops into hybrids? Scientists have figured out some of the prerequisites of hybrid breeding, e.g., the development of so-called heterotic groups of complementary inbred lines.^5^ At first glance, potato (*Solanum tuberosum* L.) may seem an unlikely target for a hybrid crop. Commercial potato varieties are self-incompatible, meaning that the development of heterotic groups of inbred lines from elite varieties is not possible. Moreover, potato is autotetraploid, combining in one nucleus four sets of freely recombining chromosomes, complicating the fixation of beneficial alleles.^6^ However, these challenges made the prospect of true-seed potato only more alluring.^7^ As there are no pure-breeding lines, farmers plant tubers that are clones of a few commercially grown varieties, some of which have been in use for decades. Since pathogen populations have long adapted to these varieties, potato yields can only be maintained by generous application of pesticides.

Sanwen Huang and colleagues were not deterred by the intricacies of potato genetics and genomics. They devised a clever combination of plant genetic resources, traditional genetics, modern genome sequencing to put the design of potato hybrids on a rational basis, fast-tracking it at the same time. Their four-step pipeline from heterozygous plant genetic resource to high-yield hybrid is described in a recent *Cell* paper by Zhang et al.^8^ and works as follows. First, Zhang et al. selected four diverse diploid clones from genebanks carrying self-compatibility factors.^9,10^ The genomes of these four clones were sequenced and the number of deleterious mutations was counted. Zhang et al. settled on two clones, named PG6359 and E86-69, to feed them into the second step of their pipeline: finding the most promising selfers. Albeit self-compatible and harboring fewer deleterious variants than others, the selected clones were still highly heterozygous, masking most recessive deleterious alleles, which would manifest themselves as weak or infertile selfed progeny. Zhang et al. scanned the genomes of selfed progeny for their selected regions where one parental allele is much more prevalent than expected by chance, pinpointing regions where the other parent carries a deleterious allele. One notable obstacle they encountered was a previously unknown large-effect deleterious allele tightly linked to the desirable *Y* allele conferring yellow, nutritional tuber flesh. Thanks to frequent recombination on the chromosome harboring both genes, Zhang et al. found progeny that carried *Y*, but lacked the deleterious allele. After 2–4 generations of selfing in the most promising inbreeders, success was ascertained by genome sequencing and highly homozygous lines were chosen for the fourth and final step. F_1_ hybrids were developed by crossing two of their custom-made inbred lines and their vigor was assessed in the field and the greenhouse. Mini-tubers harvested from F_1_ were grown in small plots. The best of them outyielded its parents by > 3-fold. More importantly, the projected per-hectare yield of the diploid hybrids (40 t/ha) was very close to that of China’s leading tetraploid potato variety Lishu 6 (45 t/ha). And to share with our readers one candid detail that is not in the paper: Dr. Huang ensured us in a lecture given at our institute that the hybrid tubers tasted most deliciously. That is an impressive achievement accomplished in a short time frame (Fig. 1); yet, there is still room for improvement: the self-compatible F_1_ hybrid developed too many fruits, compromising tuber yield. And of course, the genetic innovation has yet to reach the farmers.

The Zhang et al. paper is among the most thought-provoking plant genetics papers of the last decade. First of all, it is a breeder’s tale about reinventing potato as a hybrid seed crop. However, the Zhang et al. paper is not only a potato paper; its implications reach far beyond this species. Potato shares its ‘bad’ habits (from a breeder’s perspective) of outcrossing and high heterozygosity with many other crop species. Can we use the potato pipeline to turn other tuber crops like cassava and yam or even long-lived fruit trees into hybrid seed crops? Can genomics help find genetic resources to create new heterotic groups in established hybrid crops such as maize and rice?
